# CRISPR Reveals a Distal Super-Enhancer Required for Sox2 Expression in Mouse Embryonic Stem Cells

**DOI:** 10.1371/journal.pone.0114485

**Published:** 2014-12-08

**Authors:** Yan Li, Chloe M. Rivera, Haruhiko Ishii, Fulai Jin, Siddarth Selvaraj, Ah Young Lee, Jesse R. Dixon, Bing Ren

**Affiliations:** 1 Ludwig Institute for Cancer Research, San Diego, California, United States of America; 2 The Biomedical Sciences Graduate Program, University of California San Diego, School of Medicine, San Diego, California, United States of America; 3 Medical Scientist Training Program, University of California San Diego, School of Medicine, San Diego, California, United States of America; 4 Department of Cellular and Molecular Medicine, Institute of Genome Medicine, Moores Cancer Center, University of California San Diego, School of Medicine, San Diego, California, United States of America; National University of Singapore, Singapore

## Abstract

The pluripotency of embryonic stem cells (ESCs) is maintained by a small group of master transcription factors including Oct4, Sox2 and Nanog. These core factors form a regulatory circuit controlling the transcription of a number of pluripotency factors including themselves. Although previous studies have identified transcriptional regulators of this core network, the *cis*-regulatory DNA sequences required for the transcription of these key pluripotency factors remain to be defined. We analyzed epigenomic data within the 1.5 Mb gene-desert regions around the Sox2 gene and identified a 13kb-long super-enhancer (SE) located 100kb downstream of Sox2 in mouse ESCs. This SE is occupied by Oct4, Sox2, Nanog, and the mediator complex, and physically interacts with the Sox2 locus via DNA looping. Using a simple and highly efficient double-CRISPR genome editing strategy we deleted the entire 13-kb SE and characterized transcriptional defects in the resulting monoallelic and biallelic deletion clones with RNA-seq. We showed that the SE is responsible for over 90% of Sox2 expression, and Sox2 is the only target gene along the chromosome. Our results support the functional significance of a SE in maintaining the pluripotency transcription program in mouse ESCs.

## Introduction

Sox2 is one of the three core transcription factors (Oct4, Sox2, Nanog) responsible for maintaining ESC pluripotency. These core pluripotency factors form auto-regulatory loops and transcriptionally induce a cohort of other key pluripotency genes [1]-[3]. Besides ESCs, Sox2 is also expressed in various types of adult stem cells [4], [5]. Recently, it has been reported that Sox2-expressing cancer stem cells may drive tumor initiation and relapse in medulloblastoma and skin tumor [6], [7]. Therefore, elucidating the transcriptional regulation mechanisms of Sox2 gene is important for the understanding of both pluripotency and tumorigenesis.

Enhancers play a critical role in regulating metazoan gene transcription [8]-[11]. Recent genome-wide analyses have revealed that enhancers are very abundant in the genome. Additionally, highly specific enhancer landscapes are responsible for cellular identity as they regulate distinct transcriptional programs in different cell types [12]-[16]. Clusters of enhancer constituents that together generate a domain of MED1-, and transcription factor-binding, and H3K27ac enrichment have been identified as super-enhancers (SE) or stretch enhancers [17]-[19]. Compelling genomic evidence has predicted that SEs play a particularly important role in the control of cell identity and diseases [17]-[19], but direct functional evidence is lacking.

Until recently, validating the *in vivo* function of a putative enhancer has proven technically difficult. Reporter assays confirm that the sequence can function as an enhancer exogenously, but it does not demonstrate whether a distal target gene can be activated by the enhancer *in vivo*. Knocking out the enhancer sequence is the gold standard approach, but conventional methods using homologous recombination are inefficient and labor-intensive. The recently developed CRISPR/Cas9 system [20], [21] has proven to be a highly efficient genome editing technique and offers a promising method to validate enhancer functions *in vivo*. A recent study used CRISPR to validate the importance of a SE near GATA2 in chronic myeloid leukemia by demonstrating it is responsible for 80% of GATA2 expression [22]. With this recent advance, we have attempted to examine the role of SEs in pluripotency and normal development.

In the mouse genome, Sox2 is located within a∼1.5Mb gene desert region. It has been postulated that regulatory sequences may lie in such gene deserts and modulate gene expression over long distances [23]. In this study, we examined the gene desert near Sox2 and identified a putative distal super enhancer that is marked by active histone marks only in ESCs. This 13kb-long enhancer is occupied by multiple pluripotency factors and forms a long-distance DNA loop with the Sox2 promoter from 100kb away. Using a double-CRISPR excision strategy, we studied the *in vivo* function of this SE and demonstrated that it is responsible for over 90% of Sox2 gene expression in mouse ESCs. Our results therefore provide direct evidences on the key roles of a SE in governing ESC pluripotency.

## Methods and Materials

### Cell culture and transfection experiments

The F1 *Mus musculus castaneus* × S129/SvJae mouse ESC line (F123 line) was a gift from the laboratory of Dr. Edith Heard and was previously described [24]. The cells were cultured as described previously [25]. Importantly, cells were passaged twice on 0.1% gelatin-coated feeder-free plates before harvesting. F123 cells were plated at a density of 0.5 million/mL on 0.1% gelatin-coated feeder-free plates 24 hours before transfection. Cells were triple transfected using the Mouse ES Cell Nucleofector Kit (Lonza) and Amaxa Nucleofector with 7.5ug of each CRISPR plasmid and 5ug of pBABE-Puro. Post-transfection, cells were immediately plated on puromycin-resistant MEF feeders (GlobalStem) for recovery. 48 hours after transfection 2 µg/mL of puromycin (Sigma) was supplemented to the cell culture media for 3 days to select for transfected cells. Alkaline phosphatase staining was performed using mESC culture in the presence of MEF feeder cells with the Alkaline Phosphatase Staining kit (Stemgent). For growth curve analysis, control and mutant ES cells were plated at 1×10^5^ cells in triplicates on 12-well plates and counted for six consecutive days. The cells were passaged on day 4 by making 1∶3 dilutions. The cell numbers on day 5 and 6 were multiplied by three to adjust for the dilution.

### Design of CRISPR constructs for enhancer deletion

Target-specific CRISPR guide RNAs were designed to optimize uniqueness and have limited off-targets using an online tool (http://crispr.mit.edu/). Corresponding oligonucleotides were ordered (IDT) and subcloned into the pX330 plasmid (Addgene), expressing a human codon-optimized SpCas9 and chimeric guide RNA expression plasmid, following previously published guidelines [20]. The sequences flanking mEnh-Sox2^distal^ targeted by the CRISPR constructs are:

5′-CRISPR: GACGCTTCCGTTCTTGGAGT**AGG**

3′-CRISPR: TTGGATTCCCGACAACAAGC**TGG**

### PCR and Sanger sequencing for genotyping deletion clones

Genomic DNA was isolated from clonal CRISPR targeted F123 cell lines using a DNeasy Blood & Tissue Kit (Qiagen). To screen deletion clones, SE-spanning PCR primers were designed which flank the outside of the CRISPR sgRNAs and amplify a 13kb+ region. Given efficient CRISPR cutting and repair of DNA through non-homologous end joining, a∼420bp product is expected. For PCR, 20ng of genomic DNA was used and amplified with Q5 High-Fidelity DNA Polymerase (NEB).

Subsequently, a qPCR-based genotyping method was designed to quantify the DNA copy number of the targeted regions relative to control regions. qPCR copy number primers were designed inside the targeted region to quantify the efficiency of CRISPR deletion as creating a biallelic or monoallelic deletion. qPCR was performed with 10ng of genomic DNA in triplicate using SYBR green master mix (Roche) on the LightCycler 480 (Roche). Relative levels of DNA copy number were calculated using the ΔΔCt method with copy number normalized to GAPDH intron 1 (chromosome 6) as a control.

Finally, Sanger sequencing was used to confirm the qPCR genotyping result and further characterize the deletion junction. The ∼420bp products from PCR were TOPO-cloned using a Zero Blunt TOPO PCR cloning kit (Qiagen). Purified plasmid was Sanger sequenced (Retrogen) using the M13 F primer. Sanger sequencing data of one of the biallelic (confirmed by copy number analysis) SE deletion clone were not available because we failed to expand it upon freeze/thaw. The following equation was used to calculate deletion efficiency: Deletion frequency  =  ((# monoallelic clones) +2 * (# biallelic clones))/(2 * (# of total clones)). The following are the primers used:

*mEnh-Sox2^distal^ PCR Deletion-spanning primers*

F: CCTCAAAGTGCATCTCGTCA

R: GTCCCCTCGGCTTCTGTACT

*mEnh-Sox2^distal^ copy number qPCR primers*

F: CAAATGCGCCTCTCACTTTA

R: TTCCCAGCAGTGCTTGTATG

*qPCR copy number control primers: GAPDH intron 1 (chromosome 6)*

F: CTGGCACTGCACAAGAAGAT

R: GGGTTCCTATAAATACGGACTGC

*M13F Sanger Sequencing Primer*

F: GTTTTCCCAGTCACGACGTTGTA

### RNA isolation and allele-specific RT-qPCR

Total RNA was isolated by TRIzol (Life Tech) extraction and DNaseI (Life Tech) treated. The SuperScript III First-Strand Synthesis SuperMix for RT-qPCR kit (Life Tech) was used for synthesis of cDNA from 1ug of RNA. RT-qPCR was performed in triplicate using SYBR green master mix (Roche) on the LightCycler 480 (Roche). Relative levels of gene expression were normalized to beta actin (*Actb*). RT-qPCR primers for *Actb* are (Fwd: 5′-CTGGCTCCTAGCACCATGAAGATC; Rev: 5′-TGCTGATCCACATCTGCTGG). The primer sequences used in this study are Pou5f1 (F: 5′-GTTGGAGAAGGTGGAACCAA, R: 5′-CCAAGGTGATCCTCTTCTGC), Nanog (F: 5′-ATGCCTGCAGTTTTTCATCC, R: 5′-GAGGAAGGGCGAGGAGAG), Esrrb (F: 5′-ACATTGCCTCTGGCTACCAC, R: 5′-CGGGCAGTTGTACTCGATG), Klf4 (F: 5′-CAGGCTGTGGCAAAACCTAT, R: 5′-CGTCCCAGTCACAGTGGTAA), Kit (F: 5′-CGTGAACTCCATGTGGCTAA, R: 5′-CGTCTCCTGGCGTTCATAAT).

To assay the allele-specific expression from Sox2 locus, we used a common forward primer to measure the *Sox2* gene expression level (Sox2-F: 5′-ACATGTGAGGGCTGGACT). A universal reverse primer (R-universal: 5′-CCTTTTGAGCATTATCAGATTTTTC) is used to measure the total *Sox2* expression level. We used two different reverse primers to measure Sox2 expression from the CAST (R-CAST: CTTTTGAGCATTATCAGATTTTTCT) or 129 allele (R-129: CTTTTGAGCATTATCAGATTTTTCC).

### Strand-specific RNA-seq

PolyA+ RNA was captured from ten micrograms of total RNA using Oligo(dT)_25_ Dynabeads (Invitrogen). The resulting RNA fraction was subjected to first-strand cDNA synthesis using random primers and dT oligos in the presence of actinomycin D. Samples were cleaned up by applying the reaction mix to mini G50 columns. The eluted sample was then subjected to second-strand synthesis with DNA polymerase I, DNA ligase, and dUNTPs in the presence of RNase H. This ensures that the cDNA strand complementary to the mRNA has dNTPs and that the other strand is specifically labeled with dUNTPs. The cDNA was purified by QIA-quick columns and sheared using a Bioruptor (Diagenode). The fragmented cDNA was subsequently end repaired, A-tailed, and then ligated to Illumina TruSeq adapters. The samples were run on a 2% high-resolution agarose gel and fragments were size-selected from 200bp-450bp. The fragments were then treated with UDGase to remove the labeled strand, and mRNA complementary cDNA strand was amplified by PCR and sequenced (2×100bp) using the Illumina HiSeq2000.

### ChIP-seq

ChIP-Seq was carried out as previously described [26] with 500 µg chromatin and 5 µg antibody with the following antibodies, H3K27ac (Abcam, ab4729) and H3K4me3 (Millipore, 04-745). ChIP and input library preparation and sequencing procedures were carried out as described previously according to Illumina protocols with minor modifications (Illumina, San Diego, CA).

### Bioinformatic analysis

All the sequencing data were mapped to mm9. ChIP-seq and Hi-C data analysis were performed as described previously [27]. For RNA-seq analysis, reads were mapped using Novoalign and USeq. FPKM for known mouse genes were generated using in-house scripts. To identify signature genes, a pseudo number of 4 was added to the FPKM value of each gene and a 3-fold cutoff was used to identify significantly changed genes comparing wild type and enhancer knockout clones. CAST and 129 genotypes were generated, and strain-specific reads at CAST and 129 SNPs were separated as described previously [25]. To improve the accuracy of calling genetic variants between the two mouse strains, we also removed a subset of variants that showed inherent biases from all downstream analyses. Briefly, we generated dummy reads across each SNP/Indel and mapped them to the genome: SNPs that showed>5% bias and Indels showing>10% bias are ignored. We also checked for copy number variation across the two alleles and ignored variants that showed significant bias (5% FDR). Finally, we removed variants identified as poor heterozygous calls (5% FDR) [25]. Functional analysis of the signature genes was performed using DAVID and GSEA.

### Data availability

The raw data for ChIP-seq and RNA-seq experiments performed in this study have been uploaded to GEO with accession number (GSE60763). The following epigenomic data used in this study were published previously: H3K27ac ChIP-seq in different mouse tissues, and p300 ChIP-seq in mESCs [28] (GSE29218); ChIP-seq of Med1, Oct4, Sox2 and Nanog in mouse ES cells [19] (GSE44288); Hi-C data in mouse J1 ES cells [29] (GSE34156) and F123 ES cells [25] (GSE48592) were combined for the analysis in this study.

## Results

### Identification of a distal Sox2 super-enhancer in mouse ESCs

**Figure 1:**
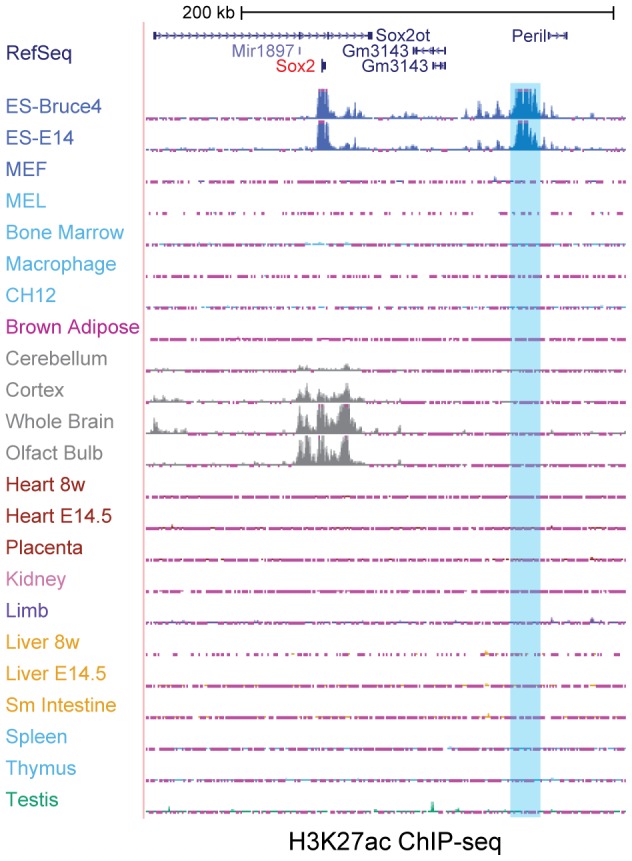
Identification of a distal mouse ESC-specific super-enhancer near Sox2 gene. In a genome-browser snapshot, 23 tracks of H3K27ac ChIP-seq data are shown. The top 2 tracks are from two different mouse ESC lines. Shadowed region shows the location of the mEnh-Sox2^distal^ enhancer.

We examined ENCODE ChIP-seq data from 23 different mouse tissues or cell types focusing on the Sox2 locus (**Fig. 1**). Using H3K27ac, a histone mark for active enhancers [30], [31], we observed a distal SE that is only present in mouse ESC lines, which is approximately 100kb downstream from the Sox2 locus. This sequence spans a relatively large 13kb region, and corresponds to a recently defined super-enhancer [19] or stretch enhancer [18]. Indeed, this region (referred to as Sox2-SE^distal^ hereafter) is one of the SEs previously identified in mouse ESCs [19]. Interestingly, although Sox2 is also expressed in several brain tissues, Sox2-SE^distal^ is not active in those tissues. Instead, a different region upstream of the Sox2 locus is marked by H3K27ac in brain tissues (**Fig. 1**), suggesting that Sox2 is regulated by different mechanisms in ESCs and neural tissues involving different *cis*-regulatory elements. Indeed a part of the region marked by H3K27ac in brain tissues has been shown to drive Sox2 expression in telencephalic neural stem cells [32].

### Epigenetic characterization of the Sox2-SE^distal^ in mouse ESCs

**Figure 2:**
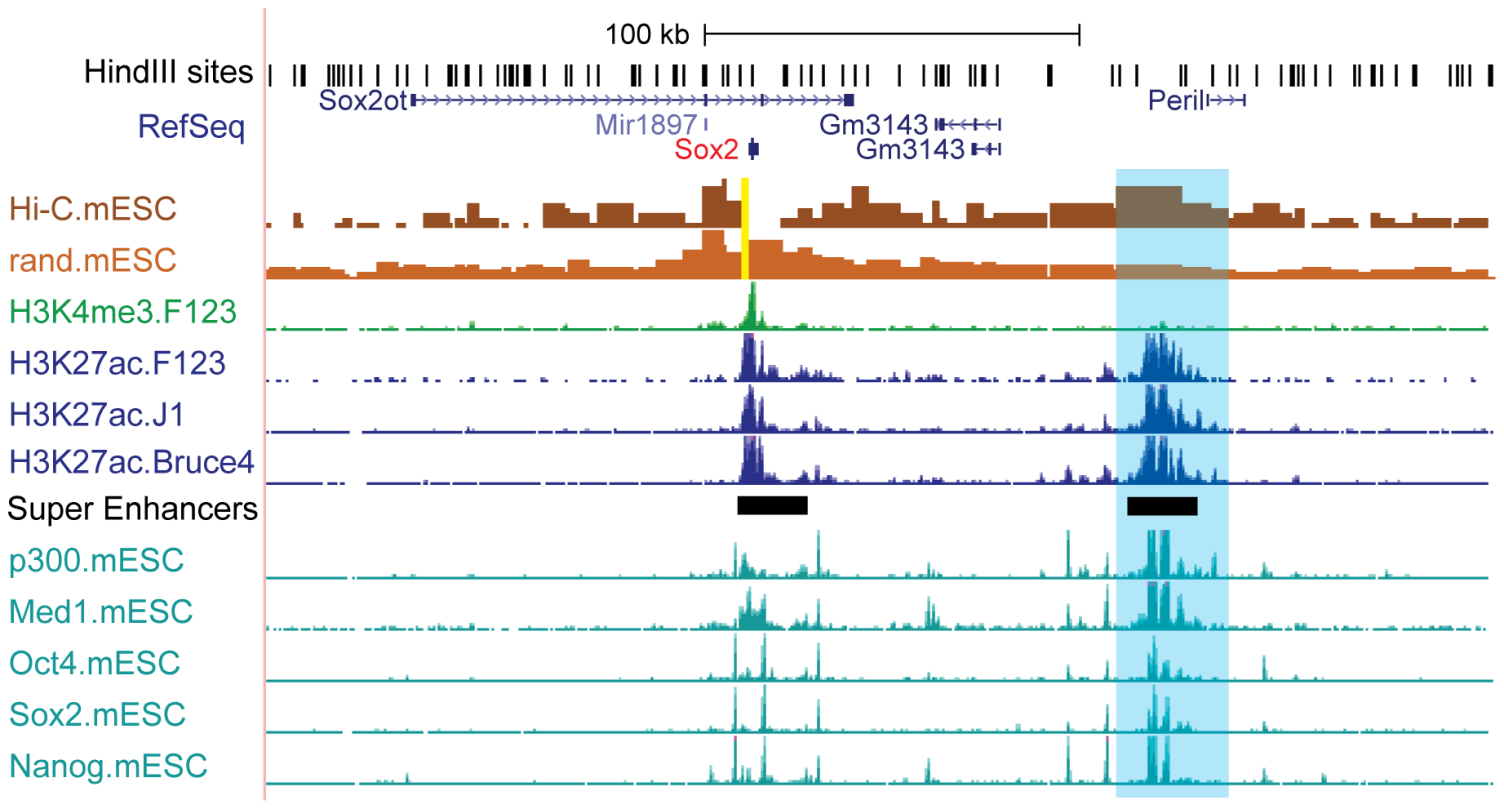
Characterization of the distal Sox2 super-enhancer. In a genome-browser snapshot, the top Hi-C track is a bar graph showing the Hi-C read number in mouse ESCs using Sox2 as the anchor (highlighted in yellow). Each bar represents one HindIII fragment between two HindIII cutting sites. The bar height represents the Hi-C read count from the anchor to the HindIII fragment. The 2^nd^ Hi-C track plots the expected value for every HindIII fragment. Therefore the blue highlighted region is a significant looping interaction from the Sox2 promoter to Sox2-SE^distal^ after comparing the top two tracks. The ChIP-seq tracks show the locations of different histone marks or protein factors in mouse ESC lines. The super-enhancer track shows the locations of two super-enhancers in mESCs identified by Hnisz et al.

We next examined the epigenetic features at Sox2-SE^distal^. First, we observed strong H3K27ac signal at this SE in two additional mouse ES cell lines (F123 and J1), indicating that usage of this SE is conserved across different mice strains. Previously published ChIP-seq data shows that Sox2-SE^distal^ is occupied by Oct4, Sox2 and Nanog (**Fig. 2**). In this study, we also observed strong binding of the transcriptional co-activator p300 and the mediator complex (Med1) (**Fig. 2**). To test if Sox2-SE^distal^ regulates Sox2 gene through long-range DNA looping, we analyzed DNA looping interactions using Hi-C data generated from mouse ESCs [27] and observed specific DNA looping signal between Sox2-SE^distal^ and the Sox2 promoter (**Fig. 2**). This long-range chromatin interaction was also observed in two other independent studies using 5C [33] and ChIA-PET [34] in mouse ESCs. Taken together, these results suggest that Sox2-SE^distal^ could be involved in the core pluripotency circuitry through regulating Sox2 gene expression.

### A simple and efficient strategy to disrupt enhancers with CRISPR

To test the *in vivo* function of Sox2-SE^distal^, we sought to delete the entire enhancer sequence from the endogenous locus. Given that the SE spans a∼13kb region, deletion of this region using conventional methods would be very inefficient. Therefore, we explored whether the recently developed CRISPR technology [20], [21] could be utilized to delete this large non-coding sequence in mouse ESCs.

**Figure 3:**
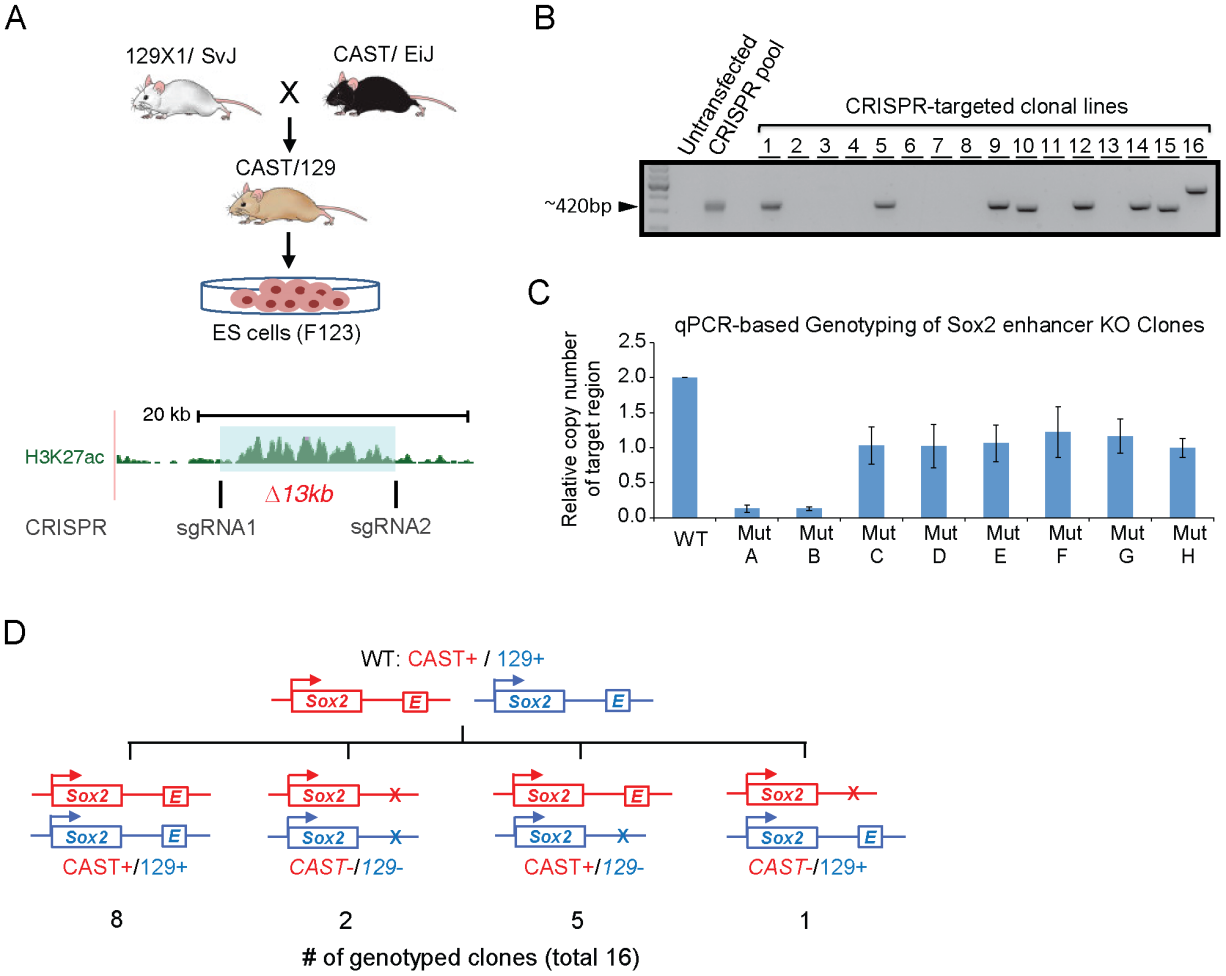
Deleting Sox2-SE^distal^ using CRISPR. (**A**) Schematic representation of CRISPR-based SE deletion strategy in a hybrid mouse ESC line, F123. Lower panel shows the location of two small guide RNA flanking the 13kb Sox2-SE^distal^. (**B**) Two PCR primers flanking (see Methods) the deleted region were used to amplify the genomic DNA from mutant clones. Successful deletion (monoallelic or biallelic) generates a small PCR product of ∼420bp with some length variation due to random mutations during non-homologous end-joining. 8 of the 16 clones show a product, meaning at least one copy of the SE is deleted. (**C**) qPCR was performed using two primers inside the SE (see Methods) to measure the copy number of the SE in WT ESCs and 8 mutant clones. Signal in WT clone is normalized to 2. Error bar: standard deviation. (**D**) Summary of the genotypes of the mutant ESC clones as confirmed by Sanger sequencing.

In the CRISPR/Cas9 system, a small guide RNA (sgRNA) targets the Cas9 protein to a specific genomic location and generates a double-strand break. Random mutations can be generated when the DNA break is repaired via non-homologous end joining (NHEJ). The efficiency of DNA cutting by the CRISPR/Cas9 system may reach over 90%, therefore it is possible to introduce multiple mutations in the same cell with high efficiency [35]. We postulated that if we introduce two sgRNAs flanking the Sox2-SE^distal^ into a cell, two DNA breaks may be generated simultaneously on the same chromosome, leading to the deletion of this long SE during DNA repair by NHEJ (**Fig. 3A**).

We therefore used this simple approach to delete Sox2-SE^distal^ in the hybrid F123 mouse ESC line (see method and materials) derived from a cross between two inbred strains, CAST and 129 [24] (**Fig. 3A**). This cell line was chosen for its high SNP density and *a priori* haplotypes. Given these characteristics, this cell line is especially amendable to robust allelic analysis of CRISPR-based deletions and downstream functional assays.

After co-transfecting two sgRNA constructs, 8 out of 16 ESC clones had the desired enhancer deletion on at least one allele (**Fig. 3B**). Copy number analysis revealed that Sox2-SE^distal^ was deleted from both alleles (biallelic clones) in 2 clones, and from one allele in the remaining 6 clones (monoallelic clones) (**Fig. 3C**). Further genotyping analysis using Sanger sequencing confirmed that among the 6 monoallelic clones, five had a deletion on the 129 allele and one on the CAST allele (**Fig. 3D** **,** **S1 Figure**). Taken together, these results suggest that our double-CRISPR strategy can delete large genomic sequences with very high efficiency (31.25%, see methods). In fact, a recent study also took a similar approach in mouse erythroleukemia cells and achieved a similar deletion frequency [36].

### Sox2-SE^distal^ is responsible for over 90% of Sox2 transcription

**Figure 4:**
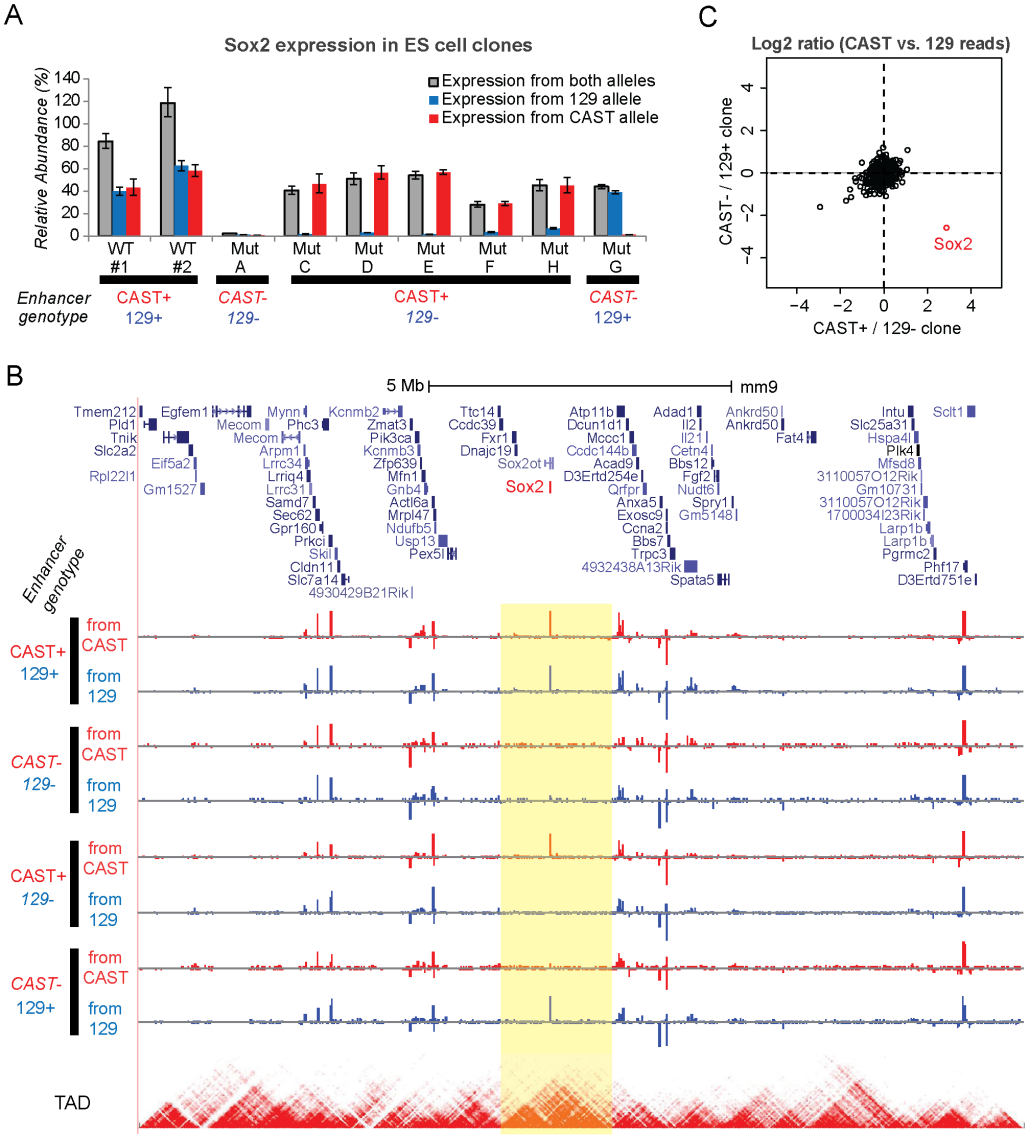
Sox2-SE^distal^ regulates Sox2 expression in *cis*. (**A**) Allele-specific RT-qPCR analysis of mESC clones with different genotypes. Samples are normalized to the average Sox2 signal between two WT biological replicates. Error bar denotes standard deviation. Note one biallelic Sox2-SE^distal^ deletion clone (Mut B from **Fig. 3**) is missing because we fail to re-expand that clone after freeze and thawing. (**B**) Genome browser tracks showing the RNA-seq signals near the Sox2 locus. Only RNA-seq reads on SNPs between CAST and 129 genomes are plotted. Red tracks: RNA-seq reads on CAST SNPs, the signal above the base line shows transcription from positive strand and the signal below the base line shows transcription from negative strand. Blue tracks: RNA-seq reads on 129 SNPs. The bottom heat map track shows TAD defined by Hi-C. The TAD containing Sox2 gene is highlighted in yellow. (**C**) Comparison of allele-specific expression from RNA-seq in the monoallelic 129 and monoallelic CAST clones. Allele specificity is defined as the log2 ratio of RNA-seq reads from CAST and 129 allele after adding a pseudo count of 10. All genes on chromosome 3 including Sox2 are plotted. Only the Sox2 gene shows CAST specific expression in CAST+/129- clone and 129 specific expression in CAST-/129+ clone, suggesting direct regulation by Sox2-SE^distal^.

We next performed allele specific RT-qPCR analysis (see methods) to quantify the effect of Sox2-SE^distal^ on Sox2 transcription. In wild type (WT) clones, both CAST and 129 alleles contribute to roughly half of total Sox2 expression (**Fig. 4A**). Strikingly, in the biallelic SE knockout clone, Sox2 expression is almost completely lost, while in monoallelic SE knockout clones, total Sox2 expression level is reduced by roughly 50% (**Fig. 4A**). Importantly, in the monoallelic clones, the level of Sox2 expression from the knockout allele is only ∼7% of the intact allele on average (**Fig. 4A**). These results reveal that a single SE, Sox2-SE^distal^, regulates transcription exclusively in *cis* and contributes to over 90% of total Sox2 expression.

In order to further test the *cis* and *trans* effects of the enhancer deletion on the transcriptome, we performed RNA-seq in four representative clones with different scenarios of enhancer deletion (WT, biallelic mutation, 129 monoallelic mutation, and CAST monoallelic mutation). Based on the sequences of RNA-seq reads and SNPs called for both parental alleles, we can determine the transcriptional outputs from the 129 or CAST allele for every gene (**Fig. 4B**). Consistent with the RT-qPCR data, the RNA-seq results also supported our conclusion that Sox2-SE^distal^ is required for Sox2 gene transcription. Importantly, none of the other genes on the same chromosomes are directly affected by Sox2-SE^distal^ deletion (**Fig. 4B–C**), suggesting Sox2 is the only target gene of Sox2-SE^distal^. It is noteworthy that Sox2 is the only active protein-coding gene sharing the same TAD [29] with Sox2-SE^distal^ (**Fig. 4B**).

### Sox2-SE^distal^ deletion alters transcription of key pluripotency genes

**Figure 5:**
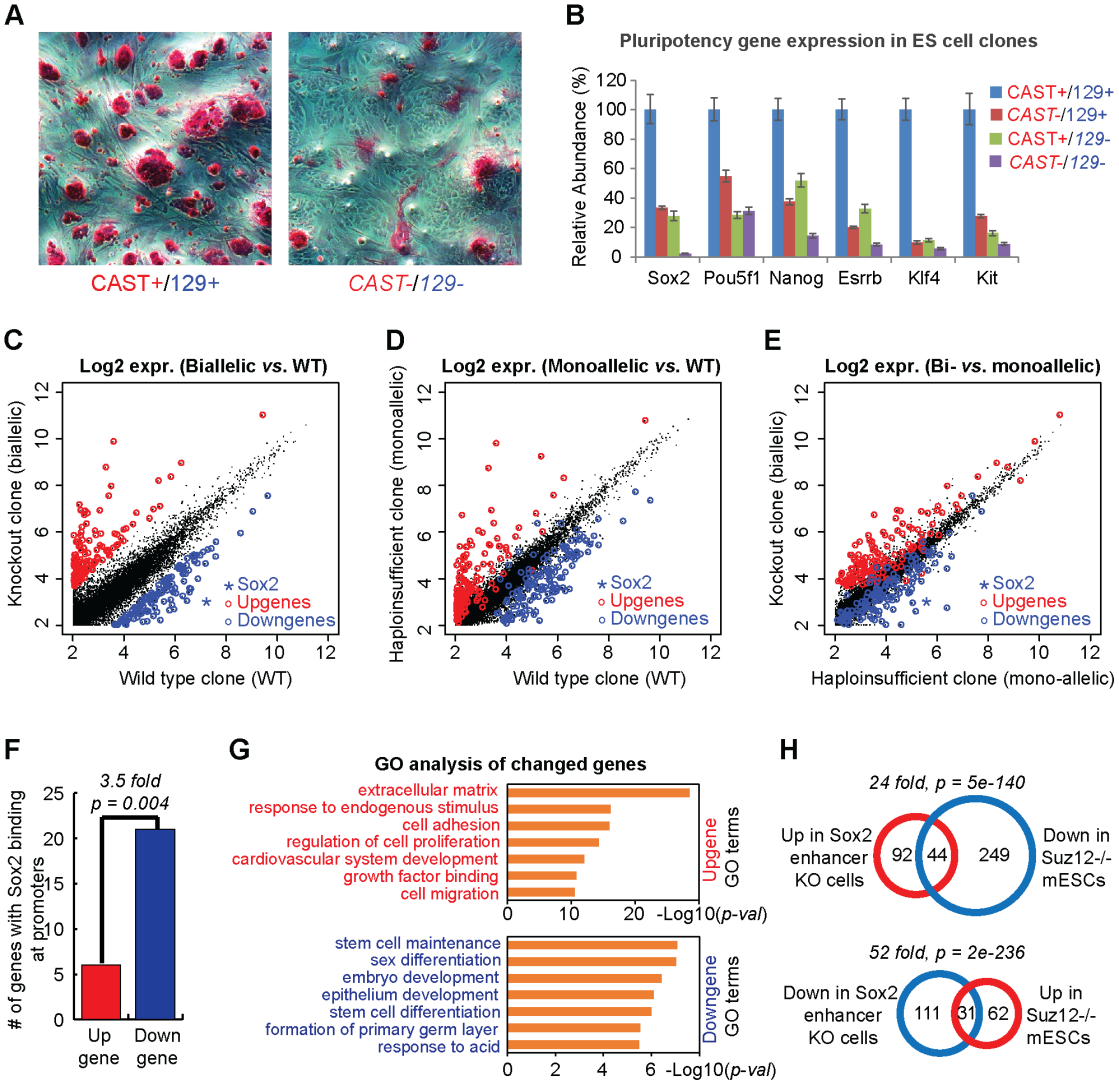
Sox2-SE^distal^ deletion affects key pluripotency genes. (**A**) Alkaline phosphatase staining of wild type (left) and biallelic Sox2-SE^distal^ deletion (right) clones. (**B**) RT-qPCR analysis of pluripotency genes in 4 representative wild type (WT) or Sox2-SE^distal^ deletion clones. (**C–E**) Scatter plots comparing the expression levels of all mouse genes in different Sox2-SE^distal^ deletion clones according to RNA-seq data. Knockout clone: biallelic Sox2-SE^distal^ mutant; haploinsufficient clone: monoallelic Sox2-SE^distal^ mutant. Plotted are log2 scaled FPKM values for each gene after adding a pseudo-value of 4. Signature up-regulated (red spots) or down-regulated (blue spots) genes were identified from the biallelic mutant clone (**C**). Expression levels of these signature genes were also compared in (**D**) and (**E**). (**F**) Bar graph comparing the up-regulated and down-regulated signature genes with Sox2 binding at promoters as called by Sox2 ChIP-seq data. P-value: Binomial test. (**G**) GO analysis identified top GO terms enriched in up-regulated or down-regulated genes upon Sox2-SE^distal^ deletion. (**H**) Venn diagrams comparing the Sox2-SE^distal^ knockout signature genes to signature genes found in Suz12 knockout mouse ESCs. P-value: binomial test.

As expected, Sox2-SE^distal^ deletion affected the proliferation and morphology of mESC culture, due to reduced expression of Sox2 and several other pluripotency factors (**Fig. 5A-B** **,** **S2 Figure**). We further analyzed the effects of Sox2-SE^distal^ deletion on the transcriptome using the RNA-seq data in different types of mutant clones (**S1 Table**). Comparing the biallelic knockout clone to the WT clone, we identified a signature of 136 up-regulated genes and 142 down-regulated genes (including Sox2) using a 3-fold cutoff (**Fig. 5C**, **S2**–**S3 Tables**). Importantly, transcription levels of most of these genes also show significant change in the monoallelic SE knockout clones (**Fig. 5D**), although to a lesser extent (**Fig. 5E**). These results suggest that partial loss of Sox2 expression also results in significant *trans* effects on gene transcription in mouse ESCs. 21 out of the 142 down-regulated genes have Sox2 binding sites at promoters, while only 6 up-regulated genes have Sox2 binding at promoters (**Fig. 5F**, **S4 Table**), suggesting some of these genes are direct targets of Sox2 transactivation. However, we cannot exclude the possibility that some genes are affected by Sox2-SE^distal^ deletion through trans-interactions or other indirect mechanisms.

Gene ontology (GO) analysis revealed distinct functions between up-regulated and down-regulated signature genes upon Sox2-SE^distal^ deletion. Consistent with the role of Sox2 as a core pluripotency factor, down-regulated signature genes are key genes involved in stem cell maintenance and early development, including Nanog, Klf4, Esrrb, Tet1, and Kit. (**Fig. 5G**). On the other hand, up-regulated signature genes are involved in late development and specialized cellular functions such as cell migration and cell communication (**Fig. 5G**).

We also performed an unbiased gene set enrichment analysis (GSEA) comparing our signature genes to hundreds of gene signatures from molecular signature database (MSigDB). Remarkably, we found that the Sox2-SE^distal^ knockout signature anti-correlates with the signature in mouse ESCs lacking the Suz12 gene [37]. Suz12 is a key component of polycomb repressive complex 2 (PRC2), which has been shown to be required for ESC differentiation [38]. Consistently, we observed a significant fraction of up-regulated signature genes that are down-regulated in Suz12-/- mouse ESCs, while the expression levels of a significant fraction of down-regulated signature genes are elevated in Suz12-/- mouse ESCs (**Fig. 5H**). It has been previously shown that Suz12 binds to a significant fraction of key developmental regulators in mESCs, and those genes are often co-occupied by Oct4, Sox2 and Nanog [39]. Our results therefore agree with a model that PRC2 antagonizes the core pluripotency factors in regulating target gene expression during differentiation.

## Discussion

We identified a mouse ESC-specific regulatory region that controls Sox2 expression through long-range DNA looping. Sox2-SE^distal^ is an exceptionally large (∼13kb) cluster of H3K27ac enrichment thereby fitting the criteria of recently defined super-enhancers or stretch enhancers [18], [19]. It has been proposed that SEs may play a key role in maintaining cell identity, and our results provide unambiguous functional data supporting this hypothesis. Interestingly, we also observed a different SE proximal to Sox2 that is also marked by histone H3K27ac exclusively in brain tissues (**Fig. 1**). This could be an SE that drives Sox2 expression in brain. It will be interesting to further explore how the Sox2 gene transitions to different enhancer usage during the development of central nervous system (CNS).

Importantly, we have demonstrated the feasibility of using a simple double-CRISPR excision strategy to delete a 13kb non-coding region (**Fig. 3A**). Because of the high efficiency of the CRISPR/Cas9 system, two double-strand breaks can be generated simultaneously on the same chromosome, leading to the loss of an entire 13kb region between the two targeting sites. The usage of a hybrid cell line (F123) also allows allele-specific measurements of enhancer activity. Upon deletion of Sox2-SE^distal^ using CRISPR, we found Sox2 expression is reduced by over 90%. As expected, Sox2-SE^distal^ deletion reduces the transcription of a number of key pluripotency genes and also induces a differentiation gene signature. We believe CRISPR will be a powerful tool for future study of *in vivo* enhancer function.

It is also striking that Sox2-SE^distal^ regulates expression of Sox2, which is located 100kb away, without affecting other nearby genes. This implies that enhancers engage in highly selective long-range interactions, and their regulation should not be simplified by assuming they regulate the nearest gene. One possible explanation for this specificity lies in the specific looping interaction between Sox2 and Sox2-SE^distal^. Our analysis of Hi-C data shows significant interaction between Sox2 and Sox2-SE^distal^ (**Fig. 2**). It is also noteworthy that, while there are other genes near Sox2-SE^distal^, only the Sox2 promoter is marked by a strong peak of H3K4me3 within the same topological domain [29], which could explain why only the Sox2 gene is targeted by Sox2-SE^distal^. A similar phenomenon was also observed in a recent study on GATA2 super-enhancer [22].

It came to our attention that a part of the region we deleted overlaps with 5′ end of the Peril ncRNA transcript [40]. As a result, the Peril transcript was lost from the alleles with Sox2-SE^distal^ deletion (data not shown). However, Sauvageau et al. did not observe significant loss of Sox2 expression in mouse embryos when they knocked out Peril. Therefore, we conclude that the effect of the Sox2-SE^distal^ deletion on Sox2 expression is not due to loss of Peril transcript.

Understanding how genomic information directs tissue-specific gene expression remains a major challenge. Identification of *cis*-regulatory elements from epigenomic information and validation by efficient genome editing techniques will be a paradigm of how to study gene regulation.

## Supporting Information

S1 Figure **Genotyping of enhancer deletion clones.** (**A**) Aligned Sanger sequencing chromatograms from deletion junctions amplified from genomic DNA of targeted Sox2-SE^distal^ clones. Two SNPs (highlighted in yellow) on the 3' end of the PCR product reveal the origin of the targeted allele. Mutant A is biallelic, Mutant G is monoallelic with the CRISPRs targeted the CAST allele, and the remaining clones are monoallelic with the CRISPRs targeting the 129 allele. (**B**) Aligned Sanger sequencing results from deletion junctions amplified from genomic DNA of targeted Sox2-SEdistal clones. Clones had varied deletions created by the Cas9 nuclease and repaired by non-homologous end joining. Mutant H contains a short inversion between the CRISPR/Cas9 cut sites consistent with the larger PCR product observed during genotyping (see Fig. 3B). The SNP at position 222 reveals the parental original of targeted allele. Mutant A is biallelic, Mutant G is monoallelic with the CRISPRs targeted the CAST allele, and the remaining clones are monoallelic with the CRISPRs targeting the 129 allele.
(TIF)

S2 Figure **Deletion of Sox2-SE^distal^ impairs ES cell proliferation.** (**A**) Western blot analysis of Sox2 gene in wild type and biallelic Sox2-SE^distal^ deletion mESC clones. (**B**) Growth curves of wild type and biallelic Sox2-SE^distal^ deletion mESC clones.
(TIF)

S1 Table **FPKM for all mouse RefSeq genes in 4 representative mESC clones from RNA-seq.**
(XLSX)

S2 Table **A list of 136 genes upregulated after deleting Sox2 enhancer.**
(XLSX)

S3 Table **A list of 142 genes downregulated after deleting Sox2 enhancer.**
(XLSX)

S4 Table **A list of genes bound by Sox2 at promoters.**
(XLSX)
