# Supplementary figures and images for: CRISPR Reveals a Distal Super-Enhancer Required for Sox2 Expression in Mouse Embryonic Stem Cells
Source: PLoS One. 2014 Dec 8;9(12):e114485. doi: 10.1371/journal.pone.0114485 (PMC4259346; doi:10.1371/journal.pone.0114485)

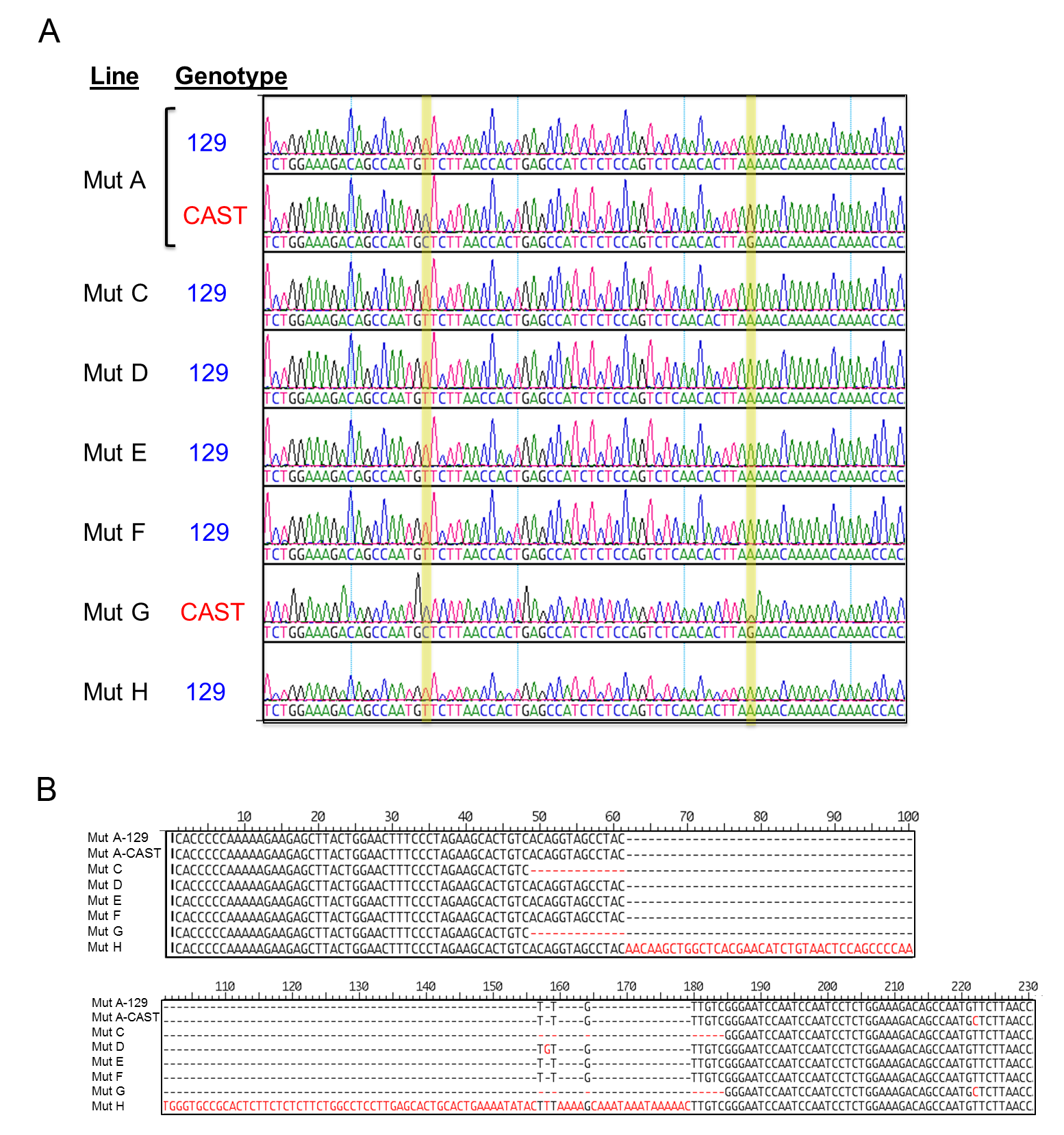

Supplement: S1 Figure — Genotyping of enhancer deletion clones. (A) Aligned Sanger sequencing chromatograms from deletion junctions amplified from genomic DNA of targeted Sox2-SEdistal clones. Two SNPs (highlighted in yellow) on the 3' end of the PCR product reveal the origin of the targeted allele. Mutant A is biallelic, Mutant G is monoallelic with the CRISPRs targeted the CAST allele, and the remaining clones are monoallelic with the CRISPRs targeting the 129 allele. (B) Aligned Sanger sequencing results from deletion junctions amplified from genomic DNA of targeted Sox2-SEdistal clones. Clones had varied deletions created by the Cas9 nuclease and repaired by non-homologous end joining. Mutant H contains a short inversion between the CRISPR/Cas9 cut sites consistent with the larger PCR product observed during genotyping (see Fig. 3B). The SNP at position 222 reveals the parental original of targeted allele. Mutant A is biallelic, Mutant G is monoallelic with the CRISPRs targeted the CAST allele, and the remaining clones are monoallelic with the CRISPRs targeting the 129 allele. (TIF) [file pone.0114485.s001.tif]

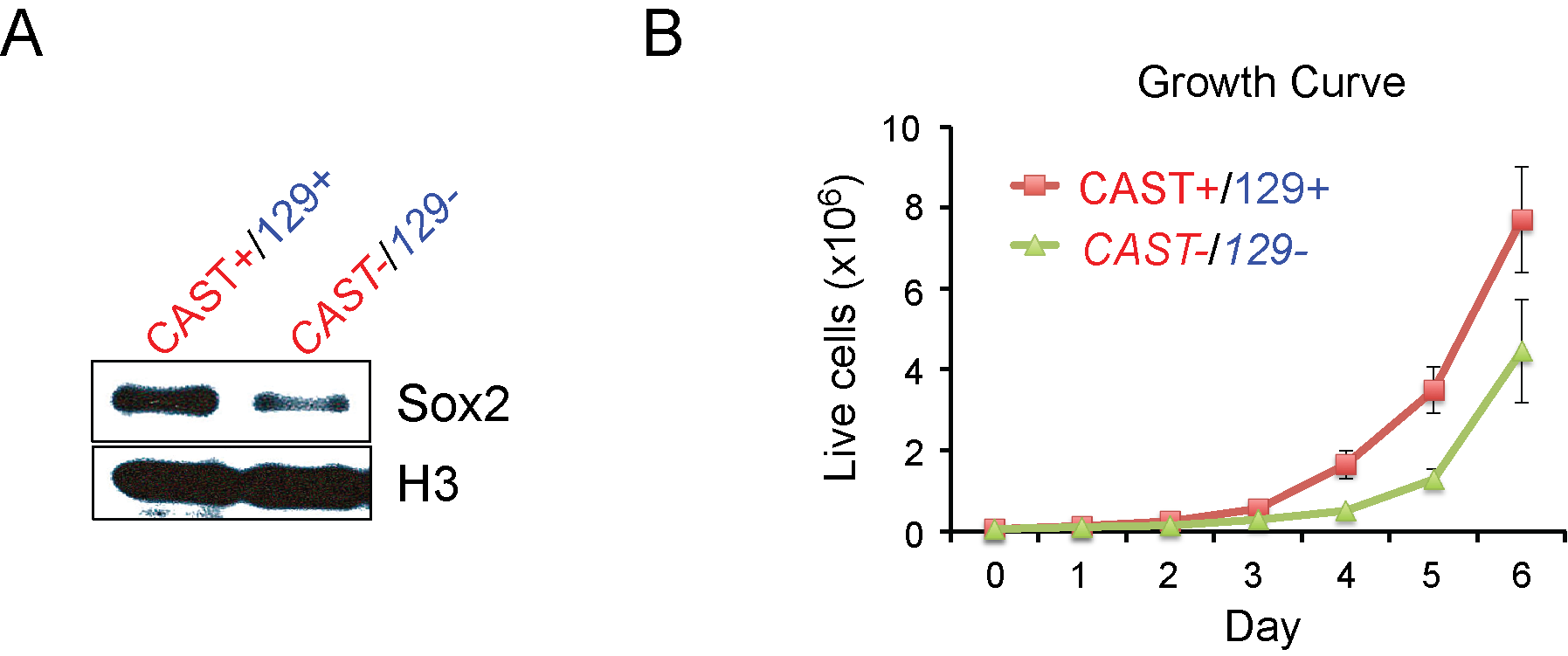

Supplement: S2 Figure — Deletion of Sox2-SEdistal impairs ES cell proliferation. (A) Western blot analysis of Sox2 gene in wild type and biallelic Sox2-SEdistal deletion mESC clones. (B) Growth curves of wild type and biallelic Sox2-SEdistal deletion mESC clones. (TIF) [file pone.0114485.s002.tif]
